# Transcultural Diabetes Nutrition Algorithm (tDNA): Venezuelan Application

**DOI:** 10.3390/nu6041333

**Published:** 2014-04-02

**Authors:** Ramfis Nieto-Martínez, Osama Hamdy, Daniel Marante, María Inés Marulanda, Albert Marchetti, Refaat A. Hegazi, Jeffrey I. Mechanick

**Affiliations:** 1 Department of Physiology, School of Medicine, Universidad Centro-Occidental “Lisandro Alvarado”, Cardio-Metabolic Unit, Barquisimeto 3001, Venezuela; E-Mail: nietoramfis@gmail.com; 2 Joslin Diabetes Center, Harvard University, Boston, MA 02215, USA; 3 Endocrinology Service, Centro Médico Docente “La Trinidad”, Caracas 1080A, Venezuela; E-Mail: daniel.marante@gmail.com; 4 Internal Medicine Department, Centro Médico “Guerra Méndez”, Valencia 2001, Venezuela; E-Mail: mariainesmarulanda@gmail.com; 5 MedERA, Inc., New York, NY 10019, USA; E-Mail: albertmarchetti@yahoo.com; 6 Department of Preventive Medicine and Community Health, University of Medicine and Dentistry of New Jersey, Newark, NJ 07101, USA; 7 Abbott Nutrition International, Columbus, OH 43219, USA; E-Mail: refaat.hegazi@abbott.com; 8 Division of Endocrinology, Diabetes, and Bone Disease, Icahn School of Medicine at Mount Sinai, New York, NY 10029, USA; E-Mail: jeffreymechanick@gmail.com

**Keywords:** diabetes, T2D, prediabetes, nutrition, MNT

## Abstract

Medical nutrition therapy (MNT) is a necessary component of comprehensive type 2 diabetes (T2D) management, but optimal outcomes require culturally-sensitive implementation. Accordingly, international experts created an evidence-based transcultural diabetes nutrition algorithm (tDNA) to improve understanding of MNT and to foster portability of current guidelines to various dysglycemic populations worldwide. This report details the development of tDNA-Venezuelan via analysis of region-specific cardiovascular disease (CVD) risk factors, lifestyles, anthropometrics, and resultant tDNA algorithmic modifications. Specific recommendations include: screening for prediabetes (for biochemical monitoring and lifestyle counseling); detecting obesity using Latin American cutoffs for waist circumference and Venezuelan cutoffs for BMI; prescribing MNT to people with prediabetes, T2D, or high CVD risk; specifying control goals in prediabetes and T2D; and describing regional differences in prevalence of CVD risk and lifestyle. Venezuelan deliberations involved evaluating typical food-based eating patterns, correcting improper dietary habits through adaptation of the Mediterranean diet with local foods, developing local recommendations for physical activity, avoiding stigmatizing obesity as a cosmetic problem, avoiding misuse of insulin and metformin, circumscribing bariatric surgery to appropriate indications, and using integrated health service networks to implement tDNA. Finally, further research, national surveys, and validation protocols focusing on CVD risk reduction in Venezuelan populations are necessary.

## 1. Introduction

At present, 346 million people worldwide have diabetes mellitus. It has been estimated that the prevalence of type 2 diabetes mellitus (T2D) will increase more in Latin America (65%) than worldwide (54%) between 2010 and 2030 [1]. In Venezuela, at least 1.7 million people suffer from T2D; and, considering only people with impaired fasting glucose, prediabetes prevalence in the country has been reported in four different regions with the number varying between 1.0% and 18.6% [2,3,4]. Moreover, the prevalence of uncontrolled T2D (A1c ≥ 7%) in Venezuela is 76%, one of the highest in Latin America [5]. In order to lessen the burden of these epidemic conditions, proper prevention and treatment interventions must be implemented.

Clinical practice guidelines (CPG) are evidence-based tools designed to assist in standardizing and improving the care of people with prediabetes and T2D [6]. Although generally useful, CPG rarely include ethnic, cultural, and/or socially-specific application cascades, which compromises their applicability and portability to diverse patient settings. In addition, the laborious task of interpreting relatively large amounts of CPG text limits their utility. In Venezuela, for example, the population is ethnically composed of Black, White, Amerindian, and mostly mixed people, geographically stratified in eight regions, with 11.2% living in rural communities [7]. As a result, there are salient differences in lifestyle and dietary habits, patterns of physical activity, food availability, medical resources, and clinical practices within the country. These issues must be considered prior to any innovative process to improve diabetes care, such as the proposed Latin America Diabetes Association (ALAD) CPG that convey culturally-adapted recommendations from various international diabetes organizations [8].

**Figure 1:**
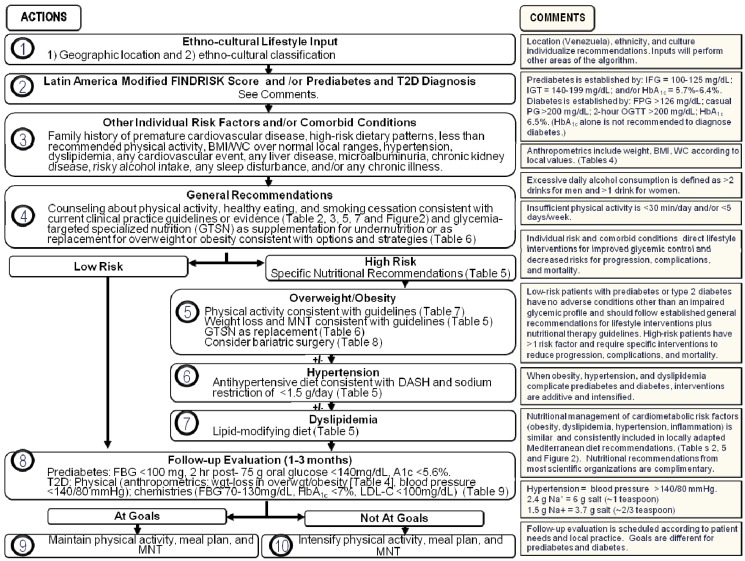
Transcultural diabetes nutrition algorithm for prediabetes and type 2 diabetes—Venezuelan Application.

Likewise, a transcultural Diabetes Nutrition Algorithm (tDNA) has been developed by an international group of expert healthcare professionals in response to the challenges mentioned above. Initially, a composite template was constructed based on extant diabetes CPG from major professional societies in North America and Europe [6]. Then, specific cultural factors that can influence the development and management of T2D were identified and a methodology created to transculturalize the template algorithm on a global scale [6]. The present report represents the current stage of tDNA adaptation in Venezuela, whereby a specific culture and locale will create its own version based on epidemiological, physiological, nutritional, and pathological parameters, as well as body composition and lifestyle characteristics that are unique to that locale. Thus, we describe herein the Venezuelan tDNA application (Figure 1) and prepare it for eventual validation protocols.

## 2. Transcultural Factors for Venezuela

Risk factors for non-communicable diseases (NCDs), both non-modifiable (genetics, gender, and age) and modifiable (improper dietary habits, sedentary lifestyle, smoking, psychological stress, and excess alcohol intake) can be expressed through metabolic/pathophysiological changes (hypertension, dyslipidemia, obesity, prediabetes, and metabolic syndrome). These processes contribute to a composite cardiovascular disease (CVD) risk. Moreover, these biologically determined factors are influenced by personal behavior, governmental policies, socio-economic factors (poverty, urbanization, and globalization), culture, politics, and both the inherent structure and unique dynamics of the target population [9]. Preventive interventions should take into account the biological characteristics and “societal risk conditions” of each population [10], justifying the need for a tDNA application.

### 2.1. Geographic, Demographic, Cultural, and Regional Characteristics of Venezuela

The Bolivarian Republic of Venezuela is a Caribbean country located in the northern section of South America. It is a federalist nation that consists of a capital district and federal agency, 23 states, and 335 municipalities. The estimated population in 2011 was 27,150,095 inhabitants, with an equal proportion of male (49.7%) and female (50.3%) residents [7]. Venezuela has experienced demographic changes that are typical for societies in economic transition. The country’s population is aging. In 2010, life expectancy at birth was 74.3 years. Sixty-six percent of the population was between 15 and 64 years old, 27.6% under 14 years, and 6.4% over 64 years [11]. Venezuela is divided into eight geographic regions: Capital, Central, Western, North-Eastern, Guayana, Andes, Zulia and the Llanos (Plains) [7]. Each region has some particularities regarding geography, climate, natural resources, population density, urban/rural proportion, food availability, and typical food and meal-based eating patterns, which can influence the recommendations for CVD and T2D prevention and treatment.

### 2.2. Non-Communicable Diseases in Latin America and Venezuela

Non-communicable diseases (NCDs), including cancer, CVD, diabetes, and chronic respiratory conditions, have been the leading cause of mortality and morbidity in the Region of the Americas, accounting for 3.9 million (77%) of a total of 5.1 million deaths in 2007. Of these 3.9 million deaths, 1.5 million (38%) were due to cardiovascular disease (CVD) and 232,000 (6%) were due to diabetes [10]. In September 2011, the United Nations General Assembly acknowledged that NCDs constitute one of the major challenges to development in the 21st century and addressed its prevention and control as a global priority [12].

Venezuelan trends in NCDs have both similarities and differences with respect to other developing countries. The Global Health Observatory Data Repository reported that in 2008 the age-standardized mortality from NCDs in Venezuela was 433 per 100,000 inhabitants, lower than the Region of the Americas (455) and total worldwide figures (573). However, in adults, aged 30–70 years, CVD- and diabetes-related mortality was 200 per 100,000, higher than the Region of the Americas (169) [13]. In the Venezuelan population older than 25 years compared with the Region, elevated fasting plasma glucose in men (11.1% *vs*. 11.5%) and women (10.9% *vs*. 9.9%) was similar, raised blood pressure in men (37.1% *vs*. 26.3%) and women (25,4% *vs*. 19.7%) was higher and obesity in men (26.6% *vs*. 23.5%) and women (34.8% *vs*. 29.7%) also was higher [13].

Latin America experienced a 14% drop in CVD between 2000 and 2009; however, this trend varied from one country to another. A study comparing the trend of coronary heart disease mortality between the years 1970 and 2000 in 10 Latin American countries found a decrease in Argentina; less pronounced decreases in Brazil, Chile, Cuba, and Puerto Rico; and increases in Mexico, Costa Rica, Ecuador, and Venezuela [14]. The absolute number of deaths due to heart disease in Venezuela rose between 2006 (24,997 deaths, 20.5% of total, 94.2 per 100.000 inhabitants) and 2009 (27,353 deaths, 20.3% of total, 94.2 per 100.000 inhabitants). Similarly, the number of deaths due to diabetes in this period increased from 7181 deaths (5.9% of total, 26.6 per 100,000 inhabitants) in 2006 to 8822 deaths (6.5% of total, 31.1 per 100,000 inhabitants) in 2009 [13].

Investigating putative risk factors responsible for CVD trends, a study conducted in four Latin American countries found that classic factors like high cholesterol, smoking, hypertension, high body mass index (BMI), and family history of coronary heart disease together accounted for 76% of cases of myocardial infarction (MI) in Venezuela [15]. These and other risk factors for MI were identified in the INTERHEART Latin American Study, including persistent psychological stress, family history of hypertension, T2D, smoking, and abdominal obesity. Abdominal obesity, smoking, and dyslipidemia were responsible for 88% of attributable MI risk. On the other hand, daily consumption of fruits and vegetables and regular exercise were associated with risk reduction [16]. Studies linking dietary habits and CVD in Venezuela have shown important disparities among different populations. In Warao Indians, for example, eating habits and intense physical activity inherent in their culture are associated with a low CVD risk profile and normal BMI, blood pressure, plasma glucose, plasma insulin, homeostatic model assessment (HOMA), uric acid, and healthy lipids, including a high HDL-C [17]. In contrast, more than 50% of the individuals in a study sample from the second largest city in Venezuela (Maracaibo) had a BMI > 25 kg/m^2^; 64% of women had central obesity (defined as waist-to-hip ratio >0.8 for women and >1 for men); 34% of men and 28% and women had high fat ingestion; 36% of men had high levels of triglycerides and VLDL-C; and 41% of women and 30% of men had decreased levels of HDL-C [18]. These differences and trends illustrate the need to incorporate complex cultural and lifestyle parameters into any therapeutic strategy intended for implementation at the population or individual level.

### 2.3. Healthcare System in Venezuela

The Region of the Americas is characterized by highly fragmented health services, which leads to restricted access, poor technical quality, irrational and inefficient use of available resources, unnecessary increases in production costs, and low user satisfaction [10]. In 2003, a Cuban Medical Mission (Misión “Barrio Adentro”) was implemented in Venezuela by agreement between Cuban and Venezuelan governments. Numerous primary care centers were established throughout the country and were staffed by Cuban doctors who were directly supervised by their own directors. Although this attempt was initially welcomed as a means to expand access, the Cuban network was kept operationally separate from the pre-existing public health structure, which ultimately increased fragmentation. Moreover, despite good intentions, these programs operated during a period of neutral and/or negative trends of NCDs deaths, cited in some of the above epidemiologic studies. Now, the physical and administrative separations between the Cuban medical mission and ordinary public health services could compromise broad implementation of tDNA. However, with government approval, the primary health network could possibly apply tDNA and increase the utilization of MNT in most social sectors of the population. This strategy recognizes that, like in many other areas of the world, healthcare coverage in private and public settings is different, necessitating different modalities of tDNA implementation.

### 2.4. Cardio-Metabolic Comorbidities and Related Risk Factors: Venezuelan Disparities by Gender, Region, Ethnicity, and Population (Urban vs. Rural)

A national population survey to investigate the prevalence of CVD and lifestyle risk factors has not been performed in Venezuela for the past 10 years, according to WHO 2012 reported statistics [13]. In order to document regional disparities in the prevalence of CVD risk factors in Venezuela, a comprehensive literature search from apparently unbiased population-based surveys and registries was conducted. The search was performed using the MEDLINE, *ScIELO*, *LILACS*, Revencyt, *BIREME*, *ScIENTI*, LIVECS and PERIóDICA bibliographic databases. Literature not indexed, such as white papers, government publications, and conference proceedings, was selected and included if considered appropriate. Studies were classified according to the size of the sampled population (all state, all city, municipalities, and smaller samples) and the studied region of the country. The prevalence of individual cardio-metabolic components in Venezuela by regions is shown in Table 1.

Two well-designed cross-sectional studies have investigated the prevalence of various cardio-metabolic risk components in specific regions of Venezuela. The first estimated the prevalence of metabolic syndrome and its association with demographic and clinical factors in 3108 subjects over 20 years of the representative state of Zulia. The age-adjusted prevalence of metabolic syndrome and atherogenic dyslipidemia was 31.2% and 24.1%, respectively. The most frequent metabolic syndrome features were low HDL-C (65%), abdominal obesity (43%), and hypertension (38%). In addition, metabolic syndrome prevalence was lower in Amerindian (17%) compared to Black (27%), White (33%) and mixed (37%) men, but no differences were found among women [3]. The study also reported that black Hispanics had worse CVD risk profiles than mixed Hispanics, with higher blood pressure, higher fasting blood glucose, increased abdominal obesity, and low HDL cholesterol [19]. The second study, CARMELA, was designed to assess the prevalence of CVD risk factors, carotid plaques, and carotid intima-media thickness in 11,150 individuals, 25–64 years old, living in seven major Latin America cities (Barquisimeto, Venezuela; Bogota, Colombia; Buenos Aires, Argentina; Lima, Peru; Mexico City, Mexico; Quito, Ecuador; and Santiago de Chile, Chile). Comparatively, Barquisimeto (Lara State in the Western region of Venezuela) had a high prevalence of metabolic syndrome (25.8%, ranked 2nd), hypertension (24.7%, ranked 2nd) and obesity (25.1%, ranked 3rd); but the prevalence was low for diabetes (6.0%, ranked 5th), hypercholesterolemia (5.7%, ranked 7th), and smoking (21.8%, ranked 7th) [20].

### 2.5. Physical Activity in Venezuela

Physical inactivity causes 5.8% of the burden of coronary heart disease and 7.2% of the burden of T2D [21]. Worldwide, 31.1% of adults are physically inactive. This number is higher in the Americas (43.3%) [22]. In Lara state (Venezuela), for example, a study in 1399 adolescents reported that only 17.3% of boys and 7.5% of girls were physically active (at least 60 min of physical activity per day on at least five days per week) [23].

Generally, throughout the country, the most popular sport is baseball, although in recent years soccer has gained popularity. Jogging and dancing, mainly in groups, have also become more prevalent. Although Venezuela is geographically diverse, differences in climate and topography have little effect on the type of physical activity that is performed. On the other hand, concerns about public safety may limit activities as crime has become more rampant in Venezuela, now among the Latin American countries with high rates of crime. In men 20–59 years old, violence was the leading cause of death, accounting for 36.5% of mortalities in 2006 and 38.2 in 2009 [13].

**Table 1:** Prevalence of adult cardio-metabolic components in eight regions of Venezuela.

Region	Obesity (%)	Diabetes (%)	Prediabetes (%)	Hypertension (%)	Dyslipidemia (%)					Metabolic Syndrome (%)	Physical Inactivity (%)	Typical Foods
					High Cholesterol	High LDL	Low HDL	High Triglycerides	Atherogenic Dyslipidemia			
Capital	35.0 U, M *^,a^ [24] 30.0 U, M *^,a^ [24]	9.5 U, M *^,a^ [25] 8.0 U, M * [26] 6.7 U, O *^,a^ [27]	9.0 U, M *^,a^ [25] 10.0 U, M * [26]	34.0 U, M * [26] 43.4 U, O *^,a^ [27]		51.6 U, O *^,a^ [27] 33.0 U, O ^a^ [28]	81.1 U, O *^,a^ [27] 56.0 U, O *^,a^ [25] 42.9 U, M *^,a^ [26] 4 4.0 U, O ^a^ [28]	43.0 U, M *^,a^ [25] 31.4 U, M * [26] 51.3 U, O *^,a^ [27] 34.0 U, O ^a^ [28]		45.5 U, O *^,a^ [27] 20.0 U, M *^,a^ [26] 37.0 U, O *^,a^ [25] 33.6 U, C *^,a^ [29]	31.5 U, O [30] Ma: 9.0, Fe: 34.0 U, O [31]	Roasted or stewed chicken, beef or fish. With rice, pasta and salad. Italian, French and Portuguese influence
Central	39.0 U, O [32]	9.0 U, O [32]		28.1 U, O [32]	59.0 U, O [32]	25.0 U, O [32]	90.0 U, O [32]	51.0 U, O [32]				
Western	25.1 U,C * [20] 26.7 U, M * [33]	6.0 U, C * [2] 11.0 U, M * [33]	15.8 U, M * [33] 1.0 U, C * [2]	23.6 U, C * [34] 24.7 U, C * [20] 29.0 UR, S * [35] 28.3 U, M * [33]	5.7 U, C * [20] 24.8 U, M * [33]	26.0 U, M * [33]	68.7 U, M * [33]	49.0 U, M * [33]	36.9 U, M * [33]	34.9 U, M * [33] 25.8 U, C * [20]		Sheep, goat and rabbit meat. Cheese and milk whey
Andeans	12.1 R, M * [30] 24.3 U, M * [33]	8.6 R, M * [30] 14.9 U, M * [33]	18.6 U, M * [30] 4.5 U, M * [33]	25.4 R, M * [36] 34.4 U, M * [33]	11.6 U, M * [33] 22.1 R, M * [36] 33.0 U, C * [37]	14.6 U, M * [33] 13.9 R, M * [36]	43.1 R, M * [36] 69.6 U, M * [33] 76.0 U, C * [37]	45.0 R, M * [36] 39.5 U, M * [33] 56.0 U, C * [37]	16.8 R, M * [36] 26.6 U, M * [33]	26.7 U, R * [30] 38.5 U, C * [37] 27.6 U, M * [38] 23.8 U, M * [33]		Potatoes, wheat and tuber. Beef, sheep and chicken meat. Fish (cultured trout). Similar to other Andean regions
Zulia	ND	Ma:7.8, Fe:7.4 S, U * [3]	Ma: 19.6, Fe: 14.9 S, U * [3]	36.9 U, C * [39]	39.3 U, O [40] Ma: 40.2, Fe: 46.0 U, O [41]		65.3 UR, S [3]	32.3 UR, S [3] 55.9 U, O [40] Ma: 47.8, Fe: 12.2 U, O [41]	26.0 UR, S * [3]	31.2 S, U * [3] 32.1 U, O [40]	71.3 UR, S * [3]	Platain (patacón), fried wheat cake
North-Eastern	ND	ND	N|D	ND	ND	ND	ND	ND	ND	ND	ND	River and sea fish, seafood, shrimp, lobster. Tuber as yam, potatoes, ocumo. Sea food rice (paella)
Guayana	ND	ND	ND	ND	ND	ND	ND	ND	ND	ND	ND	Guayanés cheese, fried fish with arepa, rice, salad and sliced plantain (tajadas)
Llanos	ND	ND	ND	ND	ND	ND	ND	ND	ND	ND	ND	Beef, deer, chiguire, turtle and lapa meat. Barbecue with cachapas, cheese and milk cream

Sample: state (S); municipality (M); city (C); other populations (O); urban (U); rural (R), (less than 2500 inhabitants); urban + rural (UR); random sample (*); Male (Ma), Female (Fe); no data (ND); abstract published in congress (^a^). Exclusion criteria included studies in children and adolescents studies with hypertension prevalence estimated by METS definition, studies including only subjects older than 60 year, studies evaluating hospitalized patients, physical activity measured by methods different than International Questionnaire of Physical Activity (IPAQ) or validated methods and studies with unclear methodology. In reference [41] and [32], data from total population were corrected.

Sadly, the actions of government to improve public safety and reduce crime have been largely ineffective. The scarcity of outdoor safety has limited opportunities to exercise regularly, as only a minority of individuals can afford or even like attending a gym. Shopping malls have become favorite places where families spend time, but activities in malls tend to be more social, sporadic, and interrupted by numerous food stands with unhealthy options. Consequently, physical activity prescribed through tDNA may be restricted to those undertakings that can be done at home or in other safe places.

### 2.6. Aspects of Nutrition in Venezuelan (Dietary Habits, Food Availability, Typical Foods, and Their Glycemic Indices)

In the United States (US) and Europe, there has been a gradual transition towards the consumption of energy-dense, high caloric foods, important drivers of overweight and obesity [42]. In Latin America, this transition has occurred faster, beginning in Venezuela in the mid-1990s when “under and over” nutrition coexisted. A similar phenomenon also exists in other areas of the world, e.g., India [43] and Pakistan, where economies are in transition. Venezuelan nutritional data is generally not published in peer review journals. However, data from surveys published by the National Statistics Institute in Venezuela have shown that caloric consumption has increased 27% from 2202 calories in 1998 to 2790 calories in 2009, which is above the minimum 2700-calories recommendation of the FAO [44]. A follow-up survey of food consumption, in which intake was calculated by household consumer purchases of certain foods (apparent consumption), reported changes between 2003 and 2010 [44]. Although there was a slight increase in the apparent consumption of legumes (8%) and fruits (12%); a greater decrease of other healthy foods such as vegetables (−20.2%) and fish (−28.4%), especially fresh fish (−43.4%) was observed. An increase of apparent consumption of meats (2.9%) especially poultry (15.8%) and a decrease of cereals (−5.9%) and roots (−9.2%) has also been noted. The survey further reported that 60% of the Venezuelan population eats three times a day and 39% eats four or five times. A study in 243 women aged 12–45 from Lara state revealed more obesity in adults (30%) than adolescents (7%), and more low weight (21%) in adolescents than adults (3%). Using 24-h recall, deficiencies in protein intake (72.0%), calories (58.1%), calcium (34.7%), zinc (20.9%), copper, (13.3%), folate (41.5%), Vitamin B6 (19.8%) and Vitamin C (62.6%) were detected. Using food frequency questionnaire, a low intake of fruits (40%) and vegetables (14%) was uncovered [45].

In Venezuela, foreign dietary habits (food acculturation) from Spain, Portugal, and Italy (Mediterranean diet) along with flavors of China and Japan have increased gastronomic variety. Whereas, indigenous foods such as “casabe” (yuca cake), “arepa” (corn flour, water and salt), and “hallaca” (a mixture of beef, pork, chicken, raisins, capers, and olives wrapped in cornmeal dough, folded within plantain leaves, tied with strings, then boiled or steamed) are still common and have remained unchanged. The national dish, called “pabellón criollo,” is a product of miscegenation and contains four basic ingredients: rice, plantain, beef, and black beans [46]. Another typical food, “empanada,” comprising fried corn cake stuffed with cheese, beans or meat, and with 322 kcal on average, is broadly consumed. In addition to its popularity, the empanada also has social importance because its sale provides economic support for many families [47]. A modified and larger version of hamburgers called “pepitos” (bread, beef or pork, sauces, fries, cheese) have become very popular in the last 30 years, mostly at dinner. “Sancocho” a soup made with vegetables and beef or hen meat, commonly provides a healthier weekend option. In addition to traditional fare, a substantial quantity of processed energy-dense fast food is also consumed in Venezuela. Indeed, 47% of all fast food eaten in the world is consumed in the Americas Region, most of it in Fast Service Restaurants [48]. Besides autochthon fast foods (arepas, empanadas, pepitos, *etc*.), many fast food chains, such as McDonalds, Burger King, Wendys, Pizza Hut, KFC, Subway, and others, are now present in Venezuela. See Table 2 for foods common to various regions of the country.

**Table 2:** Typical and recommended menus of Venezuelan foods.

Current Typical Day Menu 1		Current Typical Day Menu 2		Recommended Menu Mediterranean-Like	
**Breakfast**	**Servings**	**Breakfast**	**Servings**	**Breakfast**	**Servings**
Fried empanadas	2 units (200 g)	Fried empanadas	2 units (200 g)	Oat with low fat milk	½ cup (120 cc)
				Low fat milk	½ glass (120 cc)
Coffee with milk	1 cup	Malta	222 cc	Oat	1 spoon (7 g)
Sugar	1 spoon (12 g)			Sugar	1 spoon (12 g)
				Integral bread	2 slices (50 g)
				Goat cheese	6 spoon (30 g)
				Orange slides	1 unit (150 g)
				Natural fruit juice	1 glass (240 cc)
				Paw	1 cup (150 g)
				Sugar	½ spoon (6 g)
				Black coffee	1 cup
**Lunch**	**Servings**	**Lunch**	**Servings**	**Lunch**	**Servings**
Beef steak	210 g	Fried chicken	210 g	Black Beans soap	½ cup (180 cc)
Pasta	1 cup (170 g)	White rice	1 cup (170 g)	Meat shredded	1 cup (130 g)
Fried plantain (Tajada)	1/4 unit (75 g)	Fried plantain (Tajada)	1/4 unit (75 g)	Green (species)	¼ cup (10 g)
Banana	1 unit (200 g)	Banana	1 unit (200 g)	White rice	½ cup (100 g)
White bread	1 unit (35 g)	White bread	1 unit (35 g)	Mix salad	3 cup
Soda	1 glass (240 cc)	Natural fruit juice	1 glass (240 cc)	Tomato	1 cup (80 g)
Coffee with milk	1 unit	Melón	1 cup (150 g)	Lettuce	1 cup (80 g)
Sugar	1 spoon (12 g)	Sugar	1 spoon (12 g)	Onion	1 cup (80 g)
				Avocado	¼ unit (50 g)
				Olive Oil	2 spoon (7 cc)
				Cut fruit	1 cup (200 g)
				Melón	1 cup (150 g)
				Sugar	½ spoon (10 g)
				Snack	
				Salad fruit (Tizana)	1 cup (200 g)
**Dinner**	**Servings**	**Dinner**	**Servings**	**Dinner**	**Servings**
Arepa with white cheese	2 unit (240 g)/60 g	Arepa with white cheese	2 unit (240 g)/60 g	Mix of vegetables	2 spoon
Margarine	2 slides (600 g)	Margarine	2 slides (60 g)	Onion	1 cup (80 g)
Coffe with milk	1 cup	Soda	1 glass (240 cc)	Tomato	1 cup (80 g)
Sugar	1 spoon (12 g)			Capsicum	1 cup (170 g)
				Fish or tuna	1 slide or 1 cup (170 g)
				Arepa	1 unit (100 g)
				Whole fruit	1 cup (150 g)
**Menu Composition**		**Menu Composition**		**Menu Composition**	
**Nutrient**	**Content**	**Nutrient**	**Content**	**Nutrient**	**Content**
Energy (kcal)	2785	Energy (kcal)	3174	Energy (kcal)	1734
Carbs (g/% of Energy )	366.5/46	Carbs (g/% of Energy )	356/44	Carbs (g/% of Energy )	220.7/50
Lipid (g/% of Energy)	147.2/41	Lipid (g/% of Energy)	155/44	Lipid (g/% of Energy)	41.0 /29
Protein (g/% of Energy)	104/13	Protein (g/% of Energy)	93/12	Protein (g/% of Energy)	126.0/21
Fiber (g)	17.4	Fiber (g)	18.6	Fiber (g)	46.2
Cholesterol (mg)	277	Cholesterol (mg)	244	Cholesterol (g)	221
Sodium (mg)	1643	Sodium (mg)	1551	Sodium (mg)	839

To better understand dietary patterns, food choices, and economics in Venezuela, in 2011, the Central Bank presented results from the IV National Household Budget and Eating Habits Survey [49]. This survey reported that the most frequent breakfast choice is arepa stuffed with cheese and served with coffee. The most common lunch consists of beef steak or fried chicken served with rice or pasta and bananas, along with fruit juice or soda. Dinner is similar to breakfast with arepas stuffed with cheese served with fruit juice or coffee. One in every three people drinks soda with lunch. In addition, 81% of Venezuelans regularly consume coffee, mostly with breakfast and dinner, and 33% of coffee consumption occurs before breakfast. These figures are maintained across all social strata [49]. Considering these data, the nutritional composition of two menus representing the typical daily intake of Venezuelans is presented in Table 2 along with a recommended menu.

Although the glycemic index is a useful tool in the nutritional treatment of T2D, a 2006 study reported that only 25% of health centers in Caracas used the glycemic index for nutritional recommendations [50]. Comparing glucose excursions produced by 50 g glucose (pattern) and its equivalent in Venezuelan food content, the highest glycemic indexes were found in casabe, tapioca (yucca), bread, and potato, followed by banana, arepa, papelón (brown sugar cane), and finally pasta and black beans at the lowest end of the range. Most of the fruits had a glycemic index below 50 [51]. See Table 3.

Alcohol consumption in Venezuela is relatively high. Data from subjects older than 15 years (69% of population) have revealed that per capita consumption of pure alcohol (L/year) in Venezuela in 2004 was the highest in Latin America [52]. Using AUDIT scoring in a Venezuelan indigenous population, 87% of men were found to be problem drinkers, thus establishing one of the highest prevalence rates for problem drinking reported in the worldwide literature [53]. Consumption increased between 1961 and 1981, decreased until 2001, and stabilized between 2001 and 2005. The types of alcohol being consumed include beer (75%), spirits (24%), and wine (1%) [54].

The only study assessing salt intake in Venezuela was conducted in Táchira state (Andes Region) through chemical analysis of food portions and reported 2082 mg/day and 1472 mg/day in subjects living at higher and lower altitudes, respectively. Although this consumption is normal, methodological problems in the study and issues related to population sampling limit extrapolation of results [55]. Elevated blood pressure observed in patients with metabolic syndrome may be due to increased reactivity of blood pressure to salt intake [56].

**Table 3:** Glycemic index of selected Venezuelan foods [51].

Carbohydrate Foods	Glycemic Index	Carbohydrate Foods	Glycemic Index
Glucose	100	Fruits	
Common foods		Banana	59
Casabe	118	Papaya	50
Tapioca (yuca)	108	Pineapple	41
Bread	98	Mango	36
Arepa	74	Tangerine	36
Brown sugar cane	71	Watermelon	34
Pasta	59	Vegetables	
Legumes		Potato	93
Black beans	51	Platain	78

Glycemic index (GI) ranks carbohydrates according to their effect on blood glucose levels. High GI ≥ 70; medium GI 56–69; low GI ≤ 55.

### 2.7. Body Composition and Cardio-Metabolic Risk, Genetic, and Ethnic Particularities in Venezuelan Population

Anthropometric measurements (BMI, body fat, and waist circumference) are used to measure body composition and the risk of T2D onset and progression. The optimal cutoff point to detect abdominal obesity may vary based on genetic differences among races. In Latin American subjects, the optimal cutoff value of waist circumference to diagnose abdominal obesity, based on the power to predict excess abdominal visceral adipose tissue, has been reported to be ≥94 cm in men and ≥90 cm in women [57]. On the other hand, the accuracy of BMI in diagnosing obesity is limited. In 13,601 subjects (age 20–79.9 years) from the Third National Health and Nutrition Examination Survey of USA, a BMI between 25 and 30 kg/m^2^ may underestimate the prevalence of obesity by 50% compared with body fat measurements by bioimpedance analysis [58]. A similar assessment in 1375 Venezuelan subjects aged ≥18 years (71% women, 54% obese) reported that a BMI cutoff of ≥30 kg/m^2^ has good specificity but misses 21% of people with excess fat. Consequently, for this segment of the population, the best BMI cutoff to categorize obesity was 27.5 kg/m^2^, with a sensitivity of 89.3% (95% CI, 87–91) and a specificity of 85.4% (95% CI, 81–89) [59]. In lean adolescents, a BMI of 21 kg/m^2^, combined with a diet high in saturated fat and a low level of physical activity may be responsible for hyperinsulinemia and dyslipidemia [60]. Therefore, lower values of waist circumference and BMI must be considered in the Venezuelan tDNA application. See Table 4.

Associations between genetic polymorphisms and CVD risk in a population of the Zulia region of Venezuela have been described. The Gly482Ser polymorphism of PGC-1 gene may be associated with increased CVD risk in T2D [61]. Also, an apparent association between the G/A UCP-3 genotype with hyperglycemia, hypertension, and increased fat percentage was observed in both sexes, and with dyslipidemia in women [62]. Also in Venezuelan subjects, previous studies have shown that the coexistence of obesity and family history of diabetes may be responsible for the deficit of pancreatic insulin secretion [63]. In men, a positive family history of T2D was responsible for decreased insulin secretion measured by HOMA β cell, and disposition index (product of insulin sensitivity and β-cell function) showed that decreased insulin secretion was not a compensatory response to insulin resistance when obesity and a family history of T2D are concomitant [64]. Indeed, brothers of asymptomatic subjects with T2D showed two times more impaired fasting glucose than control subjects [65]. These findings support the importance of family history of T2D and the presence of obesity when screening for impaired glucose regulation and risk for T2D.

**Table 4:** Classification of body composition by BMI, waist circumference and disease risk for Venezuelans [57].

Category	BMI, kg/m^2^	Obesity Class	Disease Risk	
			WC: M ≤ 94 cm F ≤ 90 cm	WC: M > 94 cm F > 90 cm
Underweight	<18.5			
Normal	18.5–24.9			
Overweight	25.0–27.4		Increased	High
Obesity	27.5–34.9	I	High	Very high
	35.0–39.9	II	Very high	Very high
	≥40	III	Extremely high	Extremely high

Body mass index (BMI); female (F); male (M); waist circumference (WC).

Prediabetes screening is hampered by the relative unavailability of appropriate biochemical tests. Diabetes risk scores have become very useful to screen for impaired glucose regulation and occult T2D, the Finnish Diabetes Risk Score (FINDRISC) being the most commonly used [66]. A study to validate FINDRISC as a screening tool for people with impaired glucose regulation in Latin America was performed replacing the original cutoff point of waist circumference with the Latin American cutoff point for subjects from Bogotá (Colombia) and Barquisimeto (Venezuela). Compared with the original, the modified FINDRISC (mFR) score had similar discrimination power to identify impaired glucose regulation in men and performed better in women. The cutoff score for the mFR to screen men and women with impaired glucose regulation in Barquisimeto was >14 [67].

Considering these data, it is proposed: (1) use mFR in Latin America to define subjects requiring OGTT to diagnose prediabetes or occult diabetes; (2) use the Latin America specific cutoff point of waist circumference to detect abdominal obesity; (3) do not rule out intervention in subjects with BMI between 27.5 and 30 kg/m^2^, since this population may have greater adiposity and CVD risk; and (4) extend the intervention to all subjects with high CVD risk, not just those with prediabetes or T2D.

## 3. Current Local CPG and Proposed Recommendations

Therapeutic lifestyle changes (TLCs) are the cornerstone for promoting cardiovascular health and should include achieving a healthy weight, increasing physical activity, reducing stress, avoiding smoking, and promoting anti-atherogenic diets. Unlike drug therapy, TLCs should be promoted at all ages and at all levels of risk, across all levels of prevention. A health professionals’ follow-up study, which included more than 42,000 subjects followed for 16 years, showed that five attributes of healthy lifestyle (no smoking, body mass index <25, moderate or vigorous physical activity ≥30 min/day, moderate alcohol consumption, and healthy diet) reduced the risk of coronary heart disease by 90% in subjects not taking medication [68]. Although TLCs are simple and inexpensive interventions, they are followed by only a minority of the population. A study in 153,000 adults reported that only 3% of the population practices four healthy habits evaluated together (no smoking, healthy weight, five servings of fruits and vegetables a day, and regular physical activity) [69]. Therefore, it is incumbent on health professionals to motivate their patients to achieve TLCs, and a portable, transcultural, and easy-to-use tool such as tDNA may help.

### 3.1. Medical Nutrition Therapy (MNT) Recommendations in T2D and Co-Morbidities

Epidemiologic and clinical data indicate that weight loss through lower energy intake and increased physical activity may decrease T2D risk more than pharmacotherapy [70,71]. Inverse associations with diabetes risk have been found with increased consumption of vegetables [72,73], whole grains [74] and limited consumption of alcohol [75]. Foods with lower glycemic indices also have been associated with improved glycemic control in subjects with diabetes [76]. Recent evidence suggests that coffee consumption may also be associated with a decreased risk for T2D and several other NCDs [77]. Certain dietary patterns also have been inversely associated with T2D and CVD risk. It has been reported that adherence to a Mediterranean-type diet is associated with a lower incidence of T2D even in the absence of changes in body weight or physical activity [78]. More recently, a Mediterranean diet supplemented with extra-virgin olive oil or nuts showed benefits in primary prevention reducing the incidence of major cardiovascular events among persons at high cardiovascular risk [79]. International and national diabetes scientific associations’ (ADA/AACE, ALAD, FENADIABETES) recommendations for nutrition and our proposed adaptations are summarized in Table 5. The Mediterranean diet meets most of the requirements of these organizations, promotes control of T2D, and may contribute to the amelioration of other CVD risk factors, including dyslipidemia, obesity, high blood pressure, and inflammation [80,81]. The diet is characterized by abundant plant foods (vegetables, breads, other forms of cereals, potatoes, beans, nuts, and seeds), fresh fruit as dessert, olive oil as the principal source of fat, dairy products (principally cheese and yogurt), fish and poultry in low to moderate amounts, zero to four eggs weekly, occasional red meat, and conservative wine consumption, normally with meals [82].

**Figure 2:**
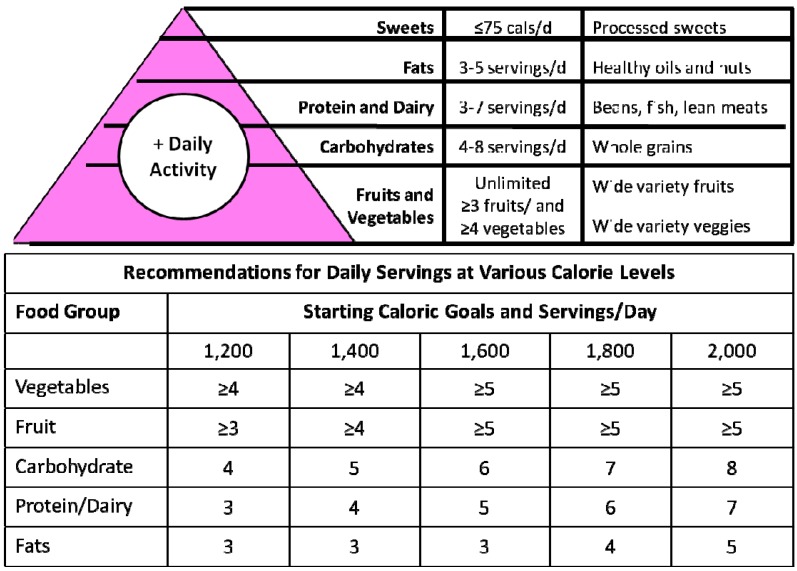
Food pyramid with Mediterranean diet recommendations and caloric goals [84].

Although a nutritionist with knowledge of MNT can help to improve outcomes in patients with T2D [83], not all patients have immediate access to their services. Therefore, it is recommended that primary care physicians be involved in the implementation of TLCs and acquire a basic knowledge of nutrition to assist patients in need. Practical implementation of dietary recommendations can be achieved using the Mediterranean food pyramid [82]. The Mayo Clinic version of the Mediterranean food pyramid also has the potential to set calorie levels by providing patients with information on the number of servings per food group that should eaten [84] (Figure 2). An example of the Mediterranean diet adapted with typical Venezuelan foods, with their compositions and portions, is presented in Table 2 as a recommendation. The implementation of tDNA in Venezuela should consider providing portable nutritional information through the Mediterranean pyramid with menus adapted to Venezuelan cultures. Any practice or expertise limitation may justify the need for consulting a nutritionist to develop additional tools and programs to support learning and implementation of behavioral changes.

**Table 5:** ADA/AACE, ALAD, FENADIABETES and proposed major nutrition recommendations for T2D.

Nutrient ^a^	ADA ^b^/AACE [85,86]	ALAD [8]	FENADIABETES [87]	Proposed recommendations
Calories	Deficit: 500–1000 kcal/day; Target: decrease weight by 5%–10% for overweight and obese individuals		Restriction: 25–30; Maintenance: 30–35	Deficit: 500–1000 kcal/day; Target: weight loss of 5% in 3 months or 10% in 6 months for overweight and obese individuals
Carbohydrate	45%–65% daily energy intake and not <130 g/day	40%–60%	45%–65%	45%–55%
Protein	15%–20% daily energy intake	15%–30%No less than <1 g/kg	0.8–1 g (80% HBV)	15%–20% 0.8%–1.2 g/kg (80% HBV)
Fat	20%–35% daily energy intake	30%–45%	25%–35%	25%–30%
Saturated fat	<7% daily energy intake	˂7%	7–10 g/day	<7% 7–10 g/day
Cholesterol	<200 mg/day		≤200 mg/day	<200 mg/day
Fiber	25–50 g/day		14 g/1000 cal	25–35 g/1000 cal (5%–10% soluble fiber)
Trans fat	Minimize or eliminate	<1%		Minimize or eliminate
Sodium ^c^	<2300 mg/day		1 g/1000 cal	1 g/1000 cal

American Association of Clinical Endocrinologists (AACE); American Diabetes Association (ADA); body mass index (BMI); high biologic value (HBV). ^a^ Apply other recommendations in subjects with dyslipidemia [88]; ^b^ ADA guidelines state that there is no optimal mix of macronutrients and therefore should be based on individualized assessment of current eating patterns, preferences, and metabolic goals; ^c^ Apply other DASH (Dietary Approaches to Stop Hypertension) recommendations in hypertensive subjects [89].

### 3.2. Diabetes-Specific Formulas to Facilitate Metabolic Control

Glycemia-targeted specialized nutrition (GTSN) contain nutrients (maltodextrin, fructose, fiber, soy protein, monounsaturated fatty acids, and antioxidants) that are designed to facilitate glycemic control in subjects with T2D and may be used as part of MNT for calorie replacement in overweight/obese subjects or supplementation for underweight individuals [6,90]. Previous evidence supports the benefits of GTSN, *i.e*., improving glycemic control and reducing chronic complications related to T2D [91,92]. A structured dietary intervention with meal replacement has also induced weight reduction in patients with diabetes [93]. For example, a study of 5145 overweight/obese subjects with T2D showed that a comprehensive lifestyle intervention, including GTSN as meal replacement, can lead to significant weight loss (*i.e*., ≥5%) and maintenance of this loss in more than 45% of patients at four years [94]. At present, no published data on the use of GTSN in Venezuela are available. While such data are gathered in local populations, recommendations drawn directly from the original tDNA template can be implemented [6]. As local data emerge and are assessed by age, disease duration, and T2D complications, perhaps more appropriate cutoff points for A1c can be established to grade glycemic control more precisely and better direct the use of GTSN [95]. See Table 6.

**Table 6:** Glycemia-targeted specialized nutrition (GTSN) for the management of prediabetes and diabetes [6].

**Overweight/Obese**	Use 2–3 GTSN ^a^ as part of a reduced calorie meal plan, as a calorie replacement for meal, partial meal or snack Calorie goals: <113 kg ^b^ = 1200 to 1500 cal; >113 kg ^b^ = 1500 to 1800 cal; Calories from GTSN. Calories from other healthy dietary source	
**Normal Weight**	Uncontrolled diabetes^ c^ A1c > 7%	1–2 GTSN per day to be incorporated into a meal plan, as a calorie replacement for meal, partial meal or snack
	Controlled diabetes ^c^ A1c < 7%	Use of GTSN should be based on clinical judgment and individual assessment ^d^
**Under weight**	Use GTSN supplements ^e^ 1–3 units/day per clinical judgment based on desired rate of weight gain and clinical tolerance	

^a^ Glycemia-targeted specialized nutrition (GTSN) are nutritional products used as calorie, partial calorie, or snack replacements in the diet. GTSNs provide approximately 100 to 300 kcal per serving; ^b^ Per Look AHEAD study (113 kg discriminator correlates with 27.5 BMI); ^c^ Glycemic targets should be individualized: Middle age and/or without complications (micro and/or macrovascular) and/or disease duration <10 year, A1c goal is 6.5%–7%. Elderly and/or complications (micro and/or macrovascular) and/or disease duration > 10 year), A1c goal is 7%–8%; ^d^ Individuals who may have muscle mass and/or function loss and/or micronutrient deficiency may benefit from GTSN supplements. Individuals who need support with weight maintenance and/or a healthy meal plan could benefit from GTSN; ^e^ GTSN supplements are complete and balanced nutritional products with ≥200 cal per serving used in addition to a typical meal plan to help promote increased nutritional intake. glycosylated hemoglobin A_1c_ (A1c).

### 3.3. Physical Activity Recommendations

Physical activity is essential in the prevention and treatment of T2D. Prospective studies in high-risk patients have concluded that regular physical activity is associated with a lower risk of developing T2D. Likewise, after T2D onset, routine physical exertion improves glucose control, slows disease progression, and prevents micro- and macrovascular complications. Benefits can be observed after a single exercise session (acute effect) improving muscular glucose uptake for 48 h and with regular ongoing exercise (chronic effect) lowering both fasting and postprandial glucose and glycated hemoglobin levels. Although aerobic exercise has been traditionally prescribed for patients with diabetes, resistance training also has benefits, augmenting both strength and insulin-sensitive muscle mass and decreasing cardiovascular risk. A combination of aerobic and resistance exercise provides the greatest benefit in the glycemic management of T2D [96].

Within Venezuela, a study in Zulia state evaluated the effectiveness of physical activity on the components of metabolic syndrome among individuals with prediabetes. In 140 subjects with mean age of 48 years, with BMI of 32.1 kg/m^2^, and who were randomly assigned to an intensive lifestyle (ILS, *n* = 70) intervention group (adapted from the U.S. Diabetes Prevention Program) or standard intervention control (*n* = 70) group for two years, significant improvements from baseline were observed in weight, waist circumference, diastolic blood pressure, triglycerides, HDL-cholesterol, fasting blood glucose, and cardiovascular health scores, ILS group *vs*. control [97]. Based on these and other similar findings [98], the physical activity recommendations in the Venezuelan tDNA include: (1) encourage at least 30 min of aerobic exercise five days/week minimally but preferably daily; (2) provide an exercise prescription that includes details for type, amount, duration, and intensity of the recommended exercise; (3) set target heart rate and perceived effort as a measure of intensity; and (4) encourage incorporating non-exercise physical activity into daily life. An exercise prescription for individuals with diabetes and prediabetes with adaptations for Venezuela are presented in Table 7.

### 3.4. Bariatric Surgery Indications

Two published studies have investigated the effect of bariatric surgery in Venezuelan patients. The first compared laparoscopic Roux-en-Y gastric bypass (RYGB) and laparoscopic sleeve gastrectomy (LSG) in 117 obese patients; no differences were found in length of stay, major complications, improvement in co-morbidities, and postoperative weight loss at one year [99]. Laparoscopic-assisted gastric banding can be considered in patients with T2D who have a BMI > 30 kg/m^2^ and laparoscopic RYGB for patients with a BMI > 35 kg/m^2^ [100]. In the second study, 15 selected patients with T2D and a BMI between 30 and 35 kg/m^2^ underwent a laparoscopic RYGB and were followed for one year. T2D remission was achieved in 93% of patients with significant drops in blood glucose and A1c. Dyslipidemia was controlled in 100% of patients and hypertension was 83.3% [101]. According to the International Diabetes Federation, bariatric surgery should be considered an alternative treatment option in patients with a BMI of 30–35 kg/m^2^ when diabetes is not adequately controlled by a medical regimen and especially when there are cardiovascular disease risk factors [100]. Recently, the American Society for Metabolic and Bariatric Surgery also noted that bariatric surgery (Gastric banding, LSG and RYBG) can benefit patients with grade 1 obesity based on evidence of clinical effectiveness, cost-effectiveness, ethics, and equity as well as the belief that this group should not be excluded from treatment [102]. Since no local bariatric surgery CPGs are available, international guidelines with minor modifications summarized in Table 8 should be considered.

**Table 7:** Exercise prescription for individuals with diabetes and prediabetes with modifications for Venezuela. Adapted from [85,98].

Type of exercise: Aerobic Resistance Stretching (Flexibility) Balance General physical activity	Intensity Level					
	Low		Medium		High	
	Aerobic	Resistance	Aerobic	Resistance	Aerobic	Resistance
	(<40% of HRmax, or <2.9 METS)	3 Big Muscle Groups	(40%–59% of HRmax, or 3.0–5.9 METS)	5 Big Muscle Groups	(≥60% of HRmax, or ≥6.0 METS)	10 Muscle Groups
Activity	Slow walking, swimming, stationary cycling, dancing	Bands (Quadriceps, biceps, triceps)	Vigorous walking, jogging, stair climbing, swimming, cycling, elliptical, fast dancing	Bands, weightlifting (dumbbells) (Quadriceps, biceps, triceps, calves, hamstrings )	Running, stair climbing, hill walking or more intense cycling, dancing, swimming	Bands, Weightlifting(dumbbells and/or gym machines)(Ten muscle groups ^a^ )
Duration	≥10 min	-	≥30 min	-	≥60 min	-
Set × reps × rest (min)	-	2 × 10 × 2	-	3 × 15 × (1–2)	-	3 × 15 × 2
Frequency	3×/week	2×/week	(3–4)×/week	3×/week	≥5×/week	(3–4)×/week
Stretching for maintaining flexibility and range of motion of joints is recommended after each exercise session. This can be achieved by passively (with the aid of the opposite limb, or by another person) or actively (using the agonist-antagonist muscle contraction).						
General physical activity: Use the stairs in the workplace. Subjects, who have practiced a sport in the past should be encouraged to take up this activity again after achieving an acceptable fitness level. Pedometer: 3000 to 7000 steps per day (individualized)						
Place: Select safer places to exercise. Walking or jogging on treadmills, stationary cycling, dancing, elliptical, bands, and weight lifting with dumbbells can be performed at home. Outdoor exercises such as jogging and cycling should be performed in organized groups. Only few options such as weight lifting using machines could require a gym.						
The initial intensity level should be selected based on age, presence of comorbidities and/or musculoskeletal limitations and fitness level of each subject. Older, less trained and/or subjects with limited mobility should start exercising at a low intensity level.						

Metabolic Equivalent (MET); Patients should be encouraged to achieve an active lifestyle and avoid sedentary living, to facilitate glycemic control and achieve health benefits. All physical activity provides some health benefits. In high CVD risk patients, exercise should be undertaken only after cardiac clearance from a physician. Patients with complications (e.g., stroke, amputation, *etc*.) will benefit from any physical activity (e.g., aerobics, resistance training, stretching) adapted to their condition and applied towards rehabilitation. In the presence of other complications of T2D (neuropathy, retinopathy, nephropathy, heart disease) exercise should be individualized; ^a^ Ten muscular groups are: quadriceps (front of legs), hamstrings (back of legs), calves, pectorals (chest), lats and trapezium (upper back), deltoids (shoulders), biceps (front of arms), triceps (back of arms), abdomen and obliques (belly) and lower back.

**Table 8:** Criteria for bariatric surgery for the management of obesity and/or diabetes [100,103].

**BMI ≥ 40 kg/m^2^**
BMI 35–39.9 kg/m^2^ and an obesity-related comorbidity, such as T2D, coronary heart disease, or severe sleep apnea.
BMI 30–34.9 kg/m^2^ under special circumstances —When diabetes is not adequately controlled by a medical regimen and especially when there are cardiovascular disease risk factors.
—Consideration may be given to laparoscopic-assisted gastric sleeve in patients with T2D who have a BMI > 30 kg/m^2^ or Roux-en-Y gastric bypass for patients with a body BMI > 35 kg/m^2^ to achieve at least short-term weight reduction. —And for each of the above: Failure to achieve and sustain weight loss after attempts at supervised lifestyle modification for at least six months; Tolerable operative risks; Understanding of operation; Commitment to treatment and long-term follow-up; Acceptance of required lifestyle changes; Diagnostic of psychiatric conditions; Suitable life expectancy.

Body mass index (BMI).

### 3.5. Follow-Up Evaluation

The effectiveness of the intervention should be assessed 1–3 months after being implemented. In this version of tDNA, monitoring indicators were specified in case of subjects with prediabetes or diabetes. Thus, the treatment must be maintained if the goals are achieved or otherwise should be intensified.

## 4. Factors Affecting TDNA Implementation

### 4.1. Changes in Food Intake, Medical Appointments, and Treatment Adherence during Holidays

Venezuela is a country where holidays are highly valued. Indeed, a large segment of the population enjoys many days off from work during Christmas, New Years, Carnival, Easter, “summer” or school vacations, and several national holidays which are extended when they occur on Thursdays or Tuesdays. Furthermore, in the past year, a new work law (Ley del Trabajo) was promulgated by the Government, making December 24 and 31 official holidays. This abundance of work-free days may be one of the reasons for the country’s high ranking on the scale of “Happiest Countries in the World”, despite current macroeconomic worries, extreme political polarization, and above all, very high rates of crime and compromised public safety. During these holidays, a generalized internal and external exodus takes place, mainly by cars and buses. Eateries are commonly associated with gasoline stations and their offerings predominantly include arepas that could contain a large amount of saturated fat. Fast food restaurant chains and tourist facilities, filled with unhealthy food choices, also abound. Moreover, holidays also encourage excessive alcohol consumption, which leads to numerous vehicular accidents and fatalities. Combined, these holiday excursions and improprieties play a significant role in the disruption of healthy lifestyles and adherence to medical advice, therapies, and healthcare visits.

### 4.2. Misconception of Obesity as an Esthetic Problem

Obesity remains one of the most difficult conditions to treat; simpler and more effective therapies are needed. Additionally, relapse is problematic because of its frequency and severity. Often a diet-resistant state emerges, in which people continue to gain weight despite persistence with their dieting. Prevailing practices for obesity include professional (empiric) and non-approved activities, ranging from simple low caloric dietary applications to use of assorted anti-obesity remedies. Furthermore, the disproportionate number of beauty pageant winners among Venezuelan women has reinforced the notion that beauty has supreme value, slimness being an inherent component of beauty, and any degree of overweightness is socially unacceptable. A large number of beauty centers have proliferated and are often staffed by untrained personnel. Significant amounts of money are spent on beauty treatments as women of all social status hope to improve their physical appearance with cosmetic procedures to plastic surgeries, ranging from botox injections to liposuction and breast implants.

This state of affairs leads to a frivolous view of obesity care that, on the one hand, inflates expectations of medical treatment yet, on the other hand, prompts abandonment of treatment for failure to achieve unrealistic goals. Disenchantment with traditional management also prompts transition to alternative and unproven therapies, most of which have more risks than benefits. Another deleterious effect of this esthetic view is that most insurance companies in Venezuela do not cover expenses for obesity treatment or anti-obesity drugs. In light of these circumstances, implementation and acceptance of a viable tDNA Venezuelan application can help to reinforce recent declarations that obesity is in fact a disease, not just a cosmetic problem [104]. In addition, based on its underlying evidence, tDNA should improve clinical outcomes. Validation of its clinical effectiveness is underway.

### 4.3. Misconceptions Surrounding Insulin

In Venezuela, the central importance of plasma insulin levels may be overstated. Expert groups have defined both metabolic syndrome and PCOS on the grounds of clinical signs or routine well-standardized laboratory measurements (e.g., plasma glucose, HDL-cholesterol, triglycerides, and blood pressure). Only the European Group for the Study of Insulin Resistance (EGIR) included measurements of plasma insulin as one of their criteria to diagnose metabolic syndrome. [105] Furthermore, plasma insulin measurement is not a standardized procedure and results may differ according to the methodology employed. Therefore, the derivation of indexes such a HOMA-IR from plasma glucose and insulin values is not warranted for clinical purposes. Also, it does not seem to be necessary to use plasma insulin measurements to establish the presence of insulin resistance. Family history; the measurement of BMI, waist circumference, distribution of body fat, triglycerides, HDL-cholesterol; and the presence of *acanthosis nigricans* provide enough information to assess insulin resistance.

Often, physicians may not be aware of the pitfalls of the use of insulin measurements and often order this test. Also, it is observed that the OGTT is performed after a “high-carb” breakfast without using the oral glucose load. In some cases, blood samples are taken every hour or every half hour with serial measurements of blood glucose, plasma insulin and even glycosuria. Since most clinical laboratories are privately owned and do not require a medical order to execute diagnostic testing, a condition exists where patients frequently seek out medical attention for “hyperinsulinism”. Many times these results are unreliable or even are not elevated.

### 4.4. Misuse of Metformin and Other Drugs in the Treatment of Obesity

Metformin is one of the most popular drugs in Venezuela. As with many drugs in the country, it is being sold without the need for a medical prescription and its price is extremely low in comparison to international prices due to price-control policies of the government. It is a common belief in Venezuela that metformin is an anti-obesity drug or that must be prescribed when plasma insulin levels are elevated. Metformin is self-prescribed and inappropriately prescribed by doctors and non-professional practitioners, and its use often supplants any attempt to initiate and maintain TLCs. A retrospective study in 924 patients attending three health centers showed heterogeneity in the indications of metformin. In one center (University Hospital), metformin was indicated only in patients with T2D. In a non-university center, metformin was used in T2D (24%) and other conditions such as insulin resistance (34%), obesity (11%), impaired glucose tolerance (3%) and PCOS (2%). Finally, in a research university setting (Pharmacology Unit), metformin was indicated in T2D (9%), insulin resistance (68%), impaired glucose tolerance (21%) and PCOS (2%) [106]. These prescribing practices may also fueled by erroneous interpretations of the Diabetes Prevention Program (DPP) study results [107] and CPGs.

Considering the factors previously mentioned, tDNA implementation is a excellent opportunity to educate physicians in order to: (a) consider obesity as a disease and regulate those individuals, professionals and non-professionals who are treating obesity only from an aesthetic point of view; (b) recommend OGTT as is standardized measuring only fasting plasma glucose levels (not insulin) and 2 h glucose after a 75 g glucose load (not after a “high carbo breakfast”) and (c) avoid prescribing metformin as an specific “anti-obesity” drug or in subjects with normal glucose regulation. A summary of current and proposed behavior/recommendations introduced as Venezuelan tDNA modifications are presented in Table 9.

**Table 9:** Summary of current status and proposed tDNA recommendations for Venezuela.

Category	Current Situations and Behaviors	Proposed tDNA Recommendations
Health system	Fragmented	Utilization of integrated health service delivery networks to educate primary care physicians to implement tDNA
Nutrition	Unbalanced diet high in calories, fat and carbohydrates. More than recommended portions of complex carbohydrates (arepas, empanadas, pepitos). High intake of saturated fat sources. Low intake of fruits and vegetables, fiber sources and fish. High intake of sodas, juices and sugar.	Primary care physicians must be involved in the implementation of TLCs and must acquire basic knowledge in MNT. Promote healthy eating consistent with current clinical practice including typical foods of each region. Promote components of the Mediterranean diets by using local foods. The main nutritional recommendations adapted to Venezuela may include: (a) Avocado and olive oil as fat source; (b) Legumes (beans as “caraotas”, peas, lentils, *etc*.) 2 or 3 times a week as the main source of carbohydrates; (c) Whole grain (oats, corn as “cachapa”, *etc*.) regularly; (d) Abundant intake of fruits (at least 3 servings, served as “Tizana”) and vegetables (at least 4 servings), anytime; (e) Avoid sugars and sweets; (f) Reduce the portions of complex carbohydrates such starch (“arepas”, rice, potatoes, pasta, *etc*.); (g) Prefer low glycemic index carbohydrates; (h) Promote soups of vegetables and chicken (“sancocho”) instead barbecues at weekends; (i) Fish (tuna, saltines) and poultry (chicken) as the main source of animal protein, at least 3 times a week and (j) Not prohibit the intake of coffee in prediabetic and diabetic subjects.
Physical activity	High rates of physical inactivity. Unsafe public areas can limit physical activity.	Prescribe physical activity recommendations that include aerobic, resistance, stretching, balance and general physical activity including previous sports practiced, suitable to be done at home and other safe places. Provide patients with an exercise prescription that includes details about the type, amount, duration, and intensity of the recommended exercises. Recommend at least 150 min per week of physical activity (advance to 300 min per week). Sports in groups (dancing, cycling, jogging) may be effective and safer. An example of a plan is presented below: a. Aerobic: 3 sessions of 30 min on 3 different days. Pause if 30 min cannot be achieved consecutively. b. Resistance: 2 sessions of 30 min. Use dumbbells or bands. Appropriate dumbbell weight or band color is allows individual to feel the effort after 15 repetitions. Perform 3 sets of 15 reps for each muscle group with one minute rest between each set. Each set plus rest are about 6 min. The initial muscle groups can be quadriceps, hamstrings, biceps, triceps and calves. Exercise of these 5 muscle groups can be done at home. Time spent to exercise all groups would be 30 min (6 min per muscle group × 5). All 10 muscle groups could be exercised in a gym. c. Stretching before and after each exercise session.
Anthropometry and body composition	Visceral obesity defined by cut-off points derived from other ethnic groups (Caucasian or Asian). BMI ≥ 30 to define Obesity.	Use the Latin America cut-off point for waist circumference to identify visceral obesity (≥94 cm in men and ≥90 cm in women ) Use a BMI ≥ 27.5 to define obesity in the Venezuelan population
Diagnosis and risk identification	T2D or prediabetes screening is not routinely done. OGTT is performed after a high-carb breakfast. In the OGTT are solicited serial measurements (every hour or half hour) of plasma glucose and insulin and even glycosuria. Measurements of plasma insulin for diagnosis of dysglycemia and/or insulin resistance.	Include the Latin America modified version of FINDRISK (mFR) as screening tool to identify people who need blood testing (OGTT) to diagnose impaired glucose regulation or occult T2D. Prediabetes and unknown diabetes is suspected by Lat mFR score > 14. Recommend an OGTT with a 75 g glucose load and not after a “high carbo breakfast”. Educate physicians to request only fasting plasma glucose and the value 2-h after 75-g of oral anhydrous glucose. Do not use plasma insulin measurements to establish the state of glucose homeostasis or insulin resistance.
Pharmacologic treatment	Metformin is used as “anti-obesity” drug and in insulin-resistance states.	Avoid prescribing metformin as an specific “anti-obesity” drug or in subjects with normal glucose regulation.
Obesity	Consideration of obesity only as a cosmetic problem.	Consider obesity as a disease and regulate those individuals, professionals and non-professionals who are treating obesity only from an aesthetic point of view.
Bariatric surgery	Offering bariatric surgery to subjects who do not meet the requirement of BMI or diabetic patients without obesity.	Recommend bariatric and metabolic surgery according to approved consensus criteria.
Other general considerations	Possible heterogeneity in the gathering and presentation of information from each country. No specific goals for Prediabetes and T2D in the main algorithm.	To standardize the information to be collected in each country in the process of transculturization of tDNA. To specify control goals for prediabetes: No progression to T2D or biochemical criteria (FBG < 100 mg, 2 h post 75 g oral glucose < 140 mg/dL, A1c < 5.7%). To specify control goals for T2D: weight goals, blood pressure < 140/80 mmHg, FBG 70–130 mg/dL, A1c < 7%, and/or LDL cholesterol < 100 mg/dL.

## 5. Conclusions

Prevention and appropriate treatment of prediabetes, T2D, and related or associated cardiovascular risk factors must be based on lifestyle changes that include an increase in physical activity and the correct implementation of MNT. Although there are clinical practice guidelines that can direct these interventions, they are not always easy to implement for their lack of portability and their failure to address the specific characteristics of the target population. The Venezuelan population, in particular, has unique epidemiological, cultural, physiological, ethnic, nutritional, pathological, and lifestyle characteristics, as well as a political, economic and social environment that is distinct and mostly unstable. All of these aspects of Venezuelan life must be considered when addressing the healthcare needs of its people, especially those needs related to food, dietary habits, and therapeutic lifestyle interventions that are so closely tied to genealogy and culture. After a group of international experts developed a diabetes-specific nutrition algorithm to facilitate and simplify the implementation of MNT for prediabetes and T2D, a local task force analyzed in detail the factors to be considered in the Venezuelan adaptation of the algorithm, which were described in this report. Recommendations from the task force are summarized in Table 9. The next steps will be to design simple and practical tools and programs to facilitate the implementation and validation of tDNA in Venezuela and to study the prevalence of cardiovascular risk factors and their relationship with the lifestyles unique to Venezuela.
